# A Comparative Analysis of Efficacy of Apatinib Combined with Transarterial Chemoembolization and Transarterial Chemoembolization Alone in the Treatment of Hepatocellular Carcinoma with Portal Vein Tumor Thrombus

**DOI:** 10.1155/2022/1255133

**Published:** 2022-03-21

**Authors:** Tao Sun, Lei Chen, Xuefeng Kan, Yanqiao Ren, Yanyan Cao, Weihua Zhang, Haohao Lu, Chuansheng Zheng

**Affiliations:** ^1^Department of Radiology, Union Hospital, Tongji Medical College, Huazhong University of Science and Technology, Wuhan, China; ^2^Hubei Province Key Laboratory of Molecular Imaging, Wuhan, China

## Abstract

**Background:**

The treatment of hepatocellular carcinoma (HCC) patients with portal vein tumor thrombus (PVTT) remains controversial due to the limited effect of sorafenib. The aim of the study was to investigate whether apatinib could improve the efficacy of transarterial chemoembolization (TACE) for patients with HCC complicated by PVTT.

**Methods:**

The study included 109 patients with HCC and PVTT who received TACE combined with apatinib (TACE + apatinib) (53 patients) or TACE alone (56 patients) between June 2015 and January 2019. Propensity score matching (PSM) analysis was used to reduce the potential selection bias. Overall survival time (OS) and time to progression (TTP) were used to evaluate the efficacy of TACE + apatinib and TACE alone.

**Results:**

Before PSM, TACE + apatinib significantly improved median TTP (7.0 vs. 3.0 months, *P* < 0.001) and median OS (15.0 vs. 7.0 months, *P* < 0.001) when compared with TACE alone. After PSM, the median TTP was significantly longer in the TACE + apatinib group, 6.0 months, than in the TACE alone group, 3.0 months (*P* < 0.001), and the median OS was significantly longer in the TACE + apatinib group, 14.0 months, than in the TACE alone group, 7.0 months (*P* < 0.001). Subgroup analysis revealed that, except for patients with Child–Pugh class B, the patients with or without extrahepatic metastases and with Child–Pugh class A had longer TTP and OS after the combined TACE + apatinib treatment than after TACE alone.

**Conclusion:**

The combination of TACE + apatinib might be an effective and safe treatment for HCC patients with PVTT.

## 1. Introduction

Hepatocellular carcinoma (HCC) is one of the most prevalent cancers worldwide and results in 782,000 deaths annually [1]. Due to latent symptoms and limited screening, most HCC patients are diagnosed at an advanced stage of the tumor [2]. Portal vein tumor thrombus (PVTT), i.e., the invasion of the main portal veins and their branches, is the most common macrovascular invasion and represents one of the significant risk factors for poor HCC prognosis [3–5]. Since patients suffering from PVTT are typically in the advanced stages of HCC [6], traditional treatment options are limited. A previous study reported that sorafenib, used as the first-line therapy for advanced HCC, benefited only modestly patients with advanced HCC complicated by PVTT [7]. A systematic review that included eight studies demonstrated that sorafenib does not provide an additional survival benefit for HCC patients with PVTT compared with other treatments such as hepatic resection, radiotherapy, and combined therapy [8].

Transarterial chemoembolization (TACE) is the standard treatment for HCC patients in the Barcelona Clinic Liver Cancer (BCLC) stage B [9]. Emerging studies suggest that TACE provides an effective and safe treatment for certain HCC patients with PVTT, especially those with preserved liver function and sufficient collateral blood flow in the vicinity of the blocked portal vein [10–12]. Based on the results of a meta-analysis, the time to progression (TTP) in patients with unresectable HCC treated by a combination of TACE with sorafenib was longer than in patients treated with TACE alone [13]. Recently, several studies documented that the combination of TACE and sorafenib as a strategy to treat HCC patients with PVTT provides greater benefits than sorafenib monotherapy [14, 15].

Apatinib is a novel molecular inhibitor selectively targeting vascular endothelial growth factor receptor-2 (VEGFR-2) tyrosine kinase. Although apatinib is similar to sorafenib, its binding affinity to VEGFR-2 is 10 times higher [16, 17]. Apatinib was approved in China for the treatment of advanced or metastatic gastric cancer [18]. Additionally, apatinib exhibited better effects against HCC than sorafenib, both in vivo and in vitro [19]. The efficacy of inhibiting HCC growth and angiogenesis by apatinib was comparable to that of sorafenib but was associated with fewer adverse effects. A single-arm, open-label phase II clinical trial reported that apatinib showed a robust clinical effect in patients with advanced HCC, with a median OS of 13.8 months and a median progression-free survival (PFS) of 8.7 months [20].

Since VEGFR-2 plays a significant role in neovascularization after TACE [21], the combination of TACE and apatinib may improve the outcome of the treatment of advanced HCC due to selective inhibition of VEGFR-2. In fact, several studies revealed that patients with advanced HCC who underwent treatment with TACE and apatinib (TACE + apatinib) benefited more than patients treated by TACE alone [21–23]. Although these results are encouraging, these studies did not directly demonstrate an improvement in the survival of HCC patients with PVTT after apatinib administration. To date, there is a shortage of data that could provide evidence for the advantages of the TACE + apatinib combination treatment. Therefore, we conducted a retrospective study to compare the efficacy and safety of TACE + apatinib with TACE alone in the treatment of HCC patients with PVTT.

## 2. Materials and Methods

### 2.1. Study Design and Patient Selection

The diagnosis of HCC was based on the European Association for the Study of the Liver (EASL) criteria [24]. Computed tomography (CT) or magnetic resonance imaging (MRI) was used to diagnose PVTT and assess its location.

This study included 109 HCC patients with PVTT who underwent TACE + apatinib or TACE alone at our hospital between June 2015 and January 2019. Among them, 53 patients were treated with TACE + apatinib and 56 with TACE alone. A propensity score matching (PSM) was used to reduce the patient selection bias and balance the variables between the two treatment groups. The treatment selection was mostly based on the patient's preference. The safety and efficacy of the TACE + apatinib treatment were analyzed retrospectively and compared with TACE alone.

Approval for this retrospective study was obtained from our college ethics committee (UHCT-IEC-SOP-016-03-01). Informed consent was obtained from the patients before the first TACE procedure. The inclusion criteria were as follows: (1) diagnosis of HCC; (2) Child–Pugh class A or B; (3) Eastern Cooperative Group Performance Status (ECOG) score of ≤2 points; (4) PVTT by contrast-enhanced CT or MRI before the TACE procedure; and (5) platelet count ≥60 × 10^9^/L. The exclusion criteria were as follows: (1) the main portal vein completely blocked without collateral circulate; (2) Child–Pugh class C; (3) liver transplantation, liver resection, or local-regional therapies before the TACE procedure; and (4) sorafenib therapy, or systemic chemotherapy.

### 2.2. TACE Procedure and Apatinib Administration

The TACE procedure and the protocol of apatinib were described in our prior studies [23, 25]. Briefly, TACE was performed by operators who had at least 5 years of experience and utilized a transfemoral arterial access route with a micropuncture system by placing a 5-F vascular introducer (Cook, Bloomington, IN, USA). Celiac and superior mesenteric arteriographies were carried out to assess the arterial anatomy, tumor supplying vessels, and the patency of the portal vein. The tumor feeding arteries were selectively cannulated with a 3-F microcatheter. A 5–20 ml aliquot of lipiodol (Lipiodol Ultraﬂuido, Guerbet, France) was mixed with 10–40 mg doxorubicin hydrochloride (Hisun Pharmaceutical Co. Ltd., Zhejiang, China) to create an emulsion, and then 5–20 ml of the emulsion was injected into tumor-feeding arteries through the microcatheter, followed by supplement embolization with gelatin sponge particles (300–700 um, Cook Medical, Bloomington, IN, USA).

In the TACE + apatinib group, apatinib was taken orally 3–5 days after each TACE procedure at an initial dosage of 500 mg/day. The dosage of apatinib was adjusted according to the patient's tolerance to the drug. The adverse events of apatinib were graded according to the National Cancer Institute Common Terminology Criteria for Adverse Events (version 4.0) [23]. If the adverse events were grade 3 or higher without effective control, the apatinib was reduced to 250 mg/day to relieve or eliminate the adverse events. If the adverse events (≥ grade 3) did not disappear after the dose adjustment, the administration of the drug was temporarily interrupted. The 250 mg/day dose was restored when the adverse events were alleviated or disappeared [23].

### 2.3. Follow-Up

All patients were followed up until January 2020. The follow-up included abdominal contrast-enhanced CT or MRI, and laboratory tests. The first follow-up was performed at the end of the fourth week after the first TACE procedure. A repeated TACE was needed when the recurrent tumor or residual lesions were found on imaging. Subsequent follow-up examinations were conducted every 2 months, starting 4 weeks after the first TACE procedure.

### 2.4. Definition and Assessments

The review of medical records included CT, MRI, and laboratory data. Tumor response was evaluated using the modified Response Evaluation Criteria in solid tumors (mRECIST) [26]. The assessment of tumor response was carried out at the first month after the first TACE procedure and then every 2 months until the time of progression or death. Disease control rate (DCR) was defined as the portion of patients who achieved a complete response (CR), partial response (PR), and stable disease (SD) (CR + PR + SD) [26]. Objective response rate was defined as the portion of patients who achieved CR, PR (CR + PR) [22]. Overall survival (OS) and time to progression (TTP) were used to evaluate the efficacy of treatment. OS was defined as the time between the first TACE and the last follow-up or all-cause death. TTP was defined as the time from the first TACE to the onset of tumor progression. Adverse events were measured based on the National Cancer Institute Common Terminology Criteria for Adverse Events, version 4.0.

### 2.5. Classification of PVTT

PVTT was classified into three types, including type I: tumor thrombus involving segmental branches of the portal vein or above; type II: tumor thrombus involving the right/left portal vein; and type III: tumor thrombus involving the main portal vein trunk based on Cheng's classification [27].

### 2.6. PSM Analysis

PSM analysis was conducted using the SPSS 24.0 software (IBM, Armonk, NY, USA). The baseline variables including age, neutrophil, lymphocyte, ALT, AST, hemoglobin, platelets, gender, HBV, type of PVTT, extrahepatic metastases, AFP, Child–Pugh, and ECOG were matched in our model. One-to-one matching without replacement was applied, and the value of the caliper was 0.2.

### 2.7. Statistical Analyses

The statistical analyses were performed using the SPSS 24.0 software. Normally distributed data, nonnormally distributed data, and categorical variables are expressed as mean ± standard deviation, median (quartile range), and frequency (percentage), respectively. The Pearson *x*^2^ test, correction *x*^2^ test, Fisher's exact test, independent-samples *t*-test, and Mann–Whitney *U* test were used to compare the two groups. Kaplan–Meier curves and log-rank test were used to compare TTP and OS between two groups. Variables with the value of *P* < 0.10 at univariate analysis were entered into univariate Cox proportional hazards regression model analysis, which was used to identify risk factors affecting OS. *P* value <0.05 (two-tailed) was considered statistically significant.

## 3. Results

### 3.1. Patient Population

**Figure 1:**
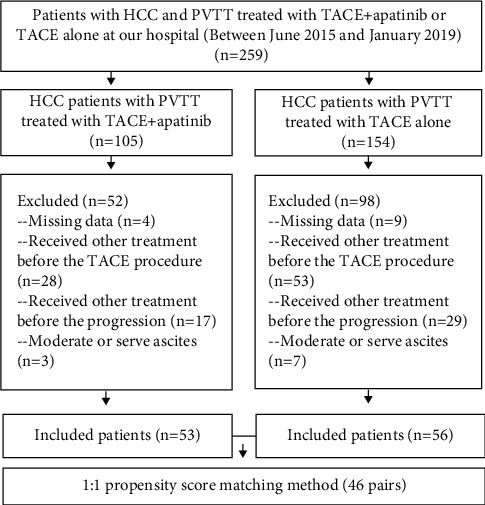
Flow chart illustrating the selection of patients. HCC, hepatocellular carcinoma; TACE, transarterial chemoembolization; TACE + apatinib, TACE combined with apatinib.

The study included 109 HCC patients with PVTT who received TACE + apatinib (*N* = 53) or TACE alone (*N* = 56). During the follow-up period, 40 (75.5%) deaths and 51 (96.2%) patients with tumor progression were observed in the TACE + apatinib group. In the TACE alone group, 52 (92.9%) deaths and 56 (100%) cases of tumor progression were observed. After the PSM analysis, 46 pairs of patients were matched (Figure 1). The baseline characteristics of the two groups did not show significant differences before and after PSM (Table 1).

### 3.2. Tumor Response

Before PSM, DCR among the patients in the TACE + apatinib group was 58.5% (31/53), a value significantly higher than in the TACE alone group (28.6%, 16/56) (*P*=0.002). The ORR in the TACE + apatinib group was 30.2% (16/53), which was significantly higher than in the TACE alone group (10.7%, 6/56) (*P*=0.011). After PSM, DCR in the TACE + apatinib group was 60.9% (28/46), which was significantly higher than in the TACE alone group (34.8%, 16/46) (*P*=0.012). The ORR among the patients in the TACE + apatinib group was 34.8% (16/46), a value significantly higher than in the TACE alone group (15.2%, 7/46) (*P*=0.030).

### 3.3. Overall Survival Analyses

**Figure 2:**
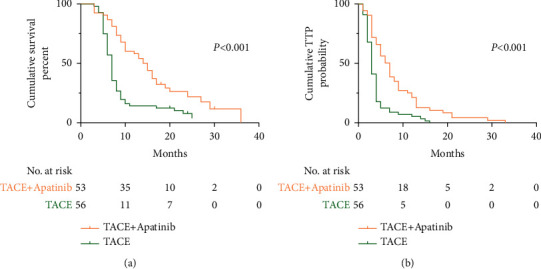
Kaplan–Meier (KM) curves for patients with hepatocellular carcinoma and portal vein tumor thrombus treated by transarterial chemoembolization (TACE) + apatinib or TACE alone. Data were analyzed before the propensity score matching. (a) KM curves for the overall survival time; (b) KM curves for the time to progression.

**Figure 3:**
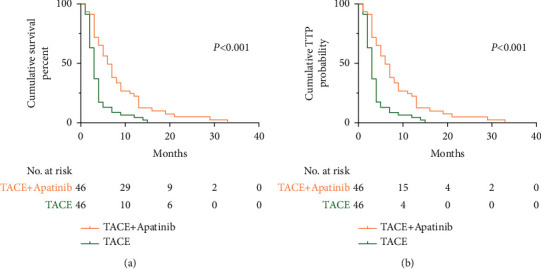
Kaplan–Meier (KM) curves for patients with hepatocellular carcinoma and portal vein tumor thrombus treated by transarterial chemoembolization (TACE) + apatinib or TACE alone. Data were analyzed after the propensity score matching. (a) KM curves for the overall survival time; (b) KM curves for the time to progression.

Before PSM, the median OS was 15.0 months in the TACE-apatinib group and 7 months in the TACE alone group (*P* < 0.001) (Figure 2(a)). After PSM, the median OS was 14.0 months in the TACE-apatinib group and 7 months in the TACE alone group (*P* < 0.001) (Figure 3(a)).

### 3.4. Time to Progression Analyses

Before PSM, the median TTP was 7.0 months in the TACE-apatinib group and 3.0 months in the TACE alone group (*P* < 0.001) (Figure 2(b)). After PSM, the median TTP was 6.0 months in the TACE-apatinib group and 3.0 months in the TACE alone group (*P* < 0.001) (Figure 3(b)). The patients in the TACE-apatinib group had a longer TTP than in the TACE alone group before and after PSM.

### 3.5. Adverse Events

A detailed list of adverse events before PSM is shown in Table 2. The adverse events of hand-foot skin reactions, hypertension, and proteinuria in the TACE-apatinib group were more common than those in the TACE group (all *P* < 0.05). However, the severe adverse events (grades ≥3) in the TACE-apatinib group were similar to those in the TACE group (all *P* < 0.05).

The adverse events associated with the TACE procedure in the two groups are shown in Table 3. Before PSM, four adverse events were observed across four patients in the TACE-apatinib group. Four adverse events were also identified across four patients in the TACE alone group.

### 3.6. Prognostic Factors Associated with TTP and OS

Univariate analysis and multivariate Cox regression analysis were performed to reveal prognostic factors for TTP and OS. The univariate analysis showed that maximal tumor size (*P* < 0.001), neutrophil-to-lymphocyte ratio (*P* < 0.001), and treatment method (TACE-apatinib or TACE alone) (*P* < 0.001) were associated with TTP and that bilirubin (*P*=0.026), maximal tumor size (*P* < 0.001), neutrophil-to-lymphocyte ratio (*P*=0.004), Child–Pugh class (*P*=0.013), and treatment method (TACE-apatinib or TACE-alone) (*P*=0.001) were associated with OS. Multivariate analysis revealed that maximal tumor size (hazard ratio (HR) = 1.170; 95% CI, 1.097–1.247; *P* < 0.001), neutrophil-to-lymphocyte ratio (HR = 1.098; 95% CI, 1.040–1.159; *P*=0.001), and TACE-apatinib (HR = 2.117; 95% CI, 1.391–3.221; *P* < 0.001) were independent protective factors for TTP and that maximal tumor size (HR = 1.293, 95% CI, 1.203–1.391; *P* < 0.001), neutrophil-to-lymphocyte ratio (HR = 1.084; 95% CI, 1.023–1.150; *P*=0.007) and TACE-apatinib (HR = 2.256; 95% CI, 1.428–3.565; *P*=0.001) were independent favorable factors for OS (Table 4).

### 3.7. Subgroup Analyses

**Figure 4:**
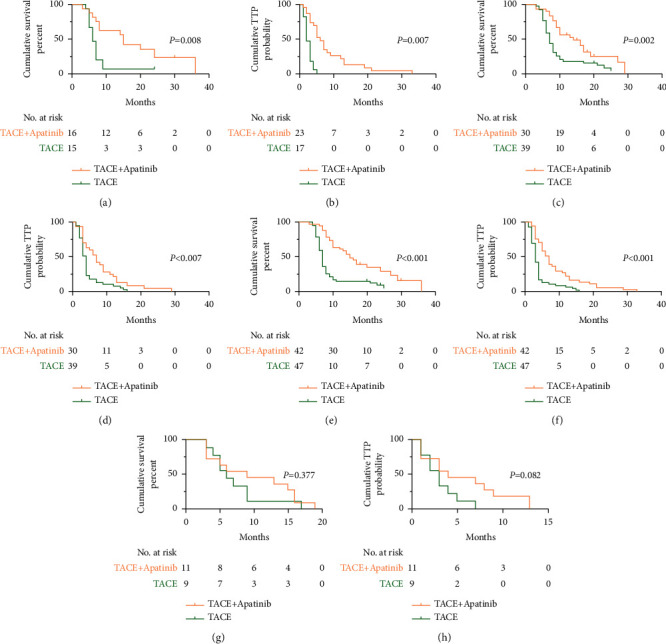
Kaplan–Meier (KM) curves for the overall survival time (OS) and time to progression (TTP) for subgroup analysis before the propensity score matching. (a, b) KM curves for OS and TTP in patients with extrahepatic metastases; (c, d) the KM curves for OS and TTP in patients without extrahepatic metastases; (e, f) KM curves for OS and TTP in patients with Child–Pugh class A; (g, h) KM curves for OS and TTP in patients with Child–Pugh B.

The results of subgroup analyses are listed in Figure 4. For the patients with or without extrahepatic metastases, with Child–Pugh class A, the median TTP and OS in the TACE-apatinib group were significantly longer than in the TACE alone group. However, for patients with Child–Pugh class B, there was no significant difference in the efficacy of the treatment between the two groups.

## 4. Discussion

Patients with PVTT usually have an unsatisfactory OS due to the aggressive course of the disease, declining liver function, high recurrence rate, and limited treatment options [28]. The median OS among HCC patients with PVTT has been reported to be as low as 2 to 4 months [28]. Although immunotherapies, e.g., atezolizumab plus bevacizumab, provided encouraging results in the treatment of unresectable HCC when compared to sorafenib, they have not been widely used clinically [29]. The adjunctive external-beam radiation therapy also showed potential in treating HCC patients with PVTT, but high-quality evidence of its benefits is yet to be obtained [30]. According to the Chinese guidelines for the treatment of HCC [31], TACE, systemic therapy (sorafenib or FOLFOX chemotherapy), surgical resection, and radiotherapy are recommended for the treatment of vascular invasion, whereas systemic therapy (sorafenib or FOLFOX4), TACE, and radiotherapy are recommended for the treatment of extrahepatic metastases. Sorafenib is recommended as the first-line therapy by the BCLC clinical staging system and sorafenib combined with TACE is more effective than TACE alone for patients with advanced HCC, but its survival benefit in HCC patients with PVTT was reported to be modest [7, 32, 33]. Considering the high cost of sorafenib treatment and its small effect, apatinib with a lower price and higher binding affinity to VEGFR-2 appears as a promising alternative for advanced HCC patients. In fact, several previous studies involving the combined treatment of TACE + apatinib reported encouraging results in patients with advanced HCC [22, 23]. Nevertheless, in these studies, cases with PVTT were only a subgroup of a broader category of patients with advanced HCC. Therefore, it is necessary to conduct studies to determine the efficacy and safety of the combined treatment of TACE + apatinib in HCC patients with PVTT.

To the best of our knowledge, only two studies focused on the effects of the combination of TACE and apatinib in the treatment of HCC patients with PVTT [22, 34]. The study conducted by Liu assessed the efficacy and tolerability of this combined treatment for advanced HCC, but it did not include TACE alone as a control group. Thus, it is essential to compare the efficacy and safety between the groups of TACE + apatinib and TACE alone using a PSM analysis, which may provide more reliable results.

The present study revealed that the combined treatment with TACE + apatinib significantly prolonged the median OS and TTP compared with TACE alone. The multivariate analysis further demonstrated that TACE + apatinib therapy was an independent factor in prolonging OS and TTP. Yuan and coworkers reported that combining TACE with sorafenib significantly increased the median OS for the HCC patients with PVTT in comparison with TACE alone (OS: 13.0 vs. 6.0 months, *P* < 0.001) [15], while Chu and colleagues reported that the median OS of patients treated with the combination of TACE and sorafenib was 12 months [24], i.e., slightly inferior to that observed in our study. A meta-analysis reported that in the included studies, the median TTP of patients treated with the combination of TACE and sorafenib ranged from 3 to 7 months [35]. These results revealed that the efficacy of TACE-apatinib in the treatment of HCC patients with PVTT was not inferior to that of TACE-sorafenib.

In our study, the side effects of apatinib were acceptable and manageable, a finding reported in many previous studies [21–23]. The common adverse events related to apatinib included hand-foot skin reactions, hypertension, diarrhea, fatigue, oral ulcer, headache, proteinuria, voice change, and gastrointestinal hemorrhage. These adverse events were predominantly grade 1 or 2. In several patients, grade 3 apatinib-related adverse events were alleviated after the dose reduction, implying that they were controllable. Generally, adverse events of apatinib in our study were similar to those reported for sorafenib [15, 24]. Thus, TACE + apatinib is safe for HCC patients with PVTT.

To further define which subgroup of patients benefited more from the combination treatment, subgroup analysis was conducted. Except for patients with Child–Pugh class B, the efficacy of TACE + apatinib for the patients with or without extrahepatic metastases and patients with Child–Pugh class A was superior to that of TACE-alone. This result indicates that the patients with these characteristics were more suitable for the treatment with the combination of TACE and apatinib. The difference in the outcomes for the patients with Child–Pugh classes A and B revealed that liver function might affect the therapeutic impact of TACE + apatinib.

Although our study provided encouraging results, it has some limitations. First, the study was designed as a retrospective analysis, and despite performing PSM, the potential patient selection bias could not be avoided entirely. Second, the data in our study originated from one clinical center. Prospective randomized controlled trials are needed in the future to provide high-level evidence for the effectiveness and safety of TACE + apatinib for the treatment of HCC patients with PVTT.

## 5. Conclusions

In conclusion, the data on the efficacy of TACE + apatinib in HCC patients with PVTT are encouraging. The TACE + apatinib combination therapy is safe in this group of patients.

## Figures and Tables

**Table 1:** The baseline characteristics of the two groups before and after PSM analysis.

Variables	TTP									OS		
	Univariable analysis			Multivariable analysis			Univariable analysis			Multivariable analysis		
	HR	95% CI	*P* value	HR	95% CI	*P* value	HR	95%CI	*P* value	HR	95% CI	*P* value
Age (years)	1.003	0.984, 1.023	0.744				1.006	0.984, 1.029	0.602			
Albumin (g/L)	0.972	0.933, 1.013	0.183				0.976	0.935, 1.020	0.281			
Bilirubin (umol/L)	1.019	0.994, 1.045	0.142				1.028	1.003, 1.053	**0.026**	1.019	0.985, 1.054	0.285
Maximal tumor size (cm)	1.169	1.097, 1.246	**<0.001**	1.170	1.097, 1.247	**<0.001**	1.284	1.196, 1.378	**<0.001**	1.293	1.203, 1.391	**<0.001**
Neutrophil-to-lymphocyte ratio	1.099	1.042, 1.160	**<0.001**	1.098	1.040, 1.159	**0.001**	1.084	1.026, 1.146	**0.004**	1.084	1.023, 1.150	**0.007**
Gender												
Male	1						1	1				
Female	1.072	0.609, 1.886	0.810				1.536	0.849, 2.779	0.156			
HBV												
Yes	1						1					
No	0.624	0.324, 1.205	0.160				0.635	0.293, 1.376	0.250			
Type of PVTT												
I	1						1					
II	1.260	0.821, 1.934	0.289				1.211	0.762, 1.926	0.417			
III	1.188	0.596, 2.369	0.624				0.756	0.342, 1.668	0.488			
Extrahepatic metastases												
Yes	1						1					
No	0.931	0.626, 1.384	0.724				1.002	0.653, 1.539	0.992			
AFP (ng/L)												
>400	1						1					
≤400	0.777	0.527, 1.147	0.205				0.827	0.546, 1.252	0.369			
Child–Pugh												
A	1						1			1		
B	1.403	0.858, 2.294	0.177				1.899	1.148, 3.142	**0.013**	1.565	0.753, 3.255	0.230
ECOG												
1	1						1					
2	1.268	0.658, 2.444	0.479				0.846	0.409, 1.751	0.652			
Treatment												
TACE + apatinib	1			1			1			1		
TACE alone	2.245	1.497, 3.365	**<0.001**	2.117	1.391, 3.221	**<0.001**	2.358	1.531, 3.632	**0.001**	2.256	1.428, 3.565	**<0.001**

PSM, propensity score matching; ECOG, Eastern Cooperative Oncology Group; HBV, hepatitis B virus; AFP, A-fetoprotein; TACE, transarterial chemoembolization; PVTT, portal vein tumor thrombus.

**Table 2:** Adverse events before PSM analysis.

Adverse events	Any grades	*P* value	≥3 grade	*P* value		
	TACE + apatinib (*n* = 53)	TACE alone (*n* = 56)		TACE + apatinib (*n* = 53)	TACE alone (*n* = 56)	
Hand-foot skin reactions	44 (83.0%)	0	≤0.001	4 (7.5%)	0	0.053
Hypertension	24 (45.3%)	5 (8.9%)	≤0.001	1 (1.9%)	0	0.486
Diarrhea	10 (18.9%)	8 (14.3%)	0.520	0	0	
Fatigue	6 (11.3%)	7 (12.5%)	0.849	0	0	
Oral ulcer	3 (5.7%)	0	0.223	0	0	
Voice change	3 (5.7%)	1 (1.8%)	0.572	1 (1.9%)	0	0.486
Proteinuria	10 (18.9%)	2 (3.6%)	0.025	1 (1.9%)	0	0.486
Gastrointestinal hemorrhage	4 (7.5%)	0	0.053	0		

TACE, transarterial chemoembolization; PSM, propensity score matching.

**Table 3:** Adverse events related to the second TACE procedure in the two groups before PSM analysis.

Adverse events	TACE + apatinib (*n* = 53)	TACE alone (*n* = 56)	*P* value
Hepatorenal syndrome	1 (1.9%)	2 (3.6%)	1.000
Inguinal hematoma	2 (3.8%)	2 (3.6%)	1.000
Hepatic arterial dissection	1 (1.9%)	0 (0%)	0.228
Pulmonary oil embolization	0 (0%)	0 (0%)	———

TACE, transarterial chemoembolization; PSM, propensity score matching.

**Table 4:** Univariate and multivariate analysis of prognostic factors for time to progression (TTP) and overall survival (OS) before the PSM analysis.

Variables	TTP									OS		
	Univariable analysis			Multivariable analysis			Univariable analysis			Multivariable analysis		
	HR	95% CI	*P* value	HR	95% CI	*P* value	HR	95% CI	*P* value	HR	95% CI	*P* value
Age (years)	1.003	0.984, 1.023	0.744				1.006	0.984, 1.029	0.602			
Albumin (g/L)	0.972	0.933, 1.013	0.183				0.976	0.935, 1.020	0.281			
Bilirubin (umol/L)	1.019	0.994, 1.045	0.142				1.028	1.003, 1.053	**0.026**	1.019	0.985, 1.054	0.285
Maximal tumor size (cm)	1.169	1.097, 1.246	**<0.001**	1.170	1.097, 1.247	**<0.001**	1.284	1.196, 1.378	**<0.001**	1.293	1.203, 1.391	**<0.001**
Neutrophil to lymphocyte ratio	1.099	1.042, 1.160	**<0.001**	1.098	1.040, 1.159	**0.001**	1.084	1.026, 1.146	**0.004**	1.084	1.023, 1.150	**0.007**
Gender												
Male	1						1	1				
Female	1.072	0.609, 1.886	0.810				1.536	0.849, 2.779	0.156			
HBV												
Yes	1						1					
No	0.624	0.324, 1.205	0.160				0.635	0.293, 1.376	0.250			
Type of PVTT												
I	1						1					
II	1.260	0.821, 1.934	0.289				1.211	0.762, 1.926	0.417			
III	1.188	0.596, 2.369	0.624				0.756	0.342, 1.668	0.488			
Extrahepatic metastases												
Yes	1						1					
No	0.931	0.626, 1.384	0.724				1.002	0.653, 1.539	0.992			
AFP (ng/L)												
>400	1						1					
≤400	0.777	0.527, 1.147	0.205				0.827	0.546, 1.252	0.369			
Child–Pugh												
A	1						1			1		
B	1.403	0.858, 2.294	0.177				1.899	1.148, 3.142	**0.013**	1.565	0.753, 3.255	0.230
ECOG												
1	1						1					
2	1.268	0.658, 2.444	0.479				0.846	0.409, 1.751	0.652			
Treatment												
TACE + apatinib	1			1			1			1		
TACE alone	2.245	1.497, 3.365	**<0.001**	2.117	1.391, 3.221	**<0.001**	2.358	1.531, 3.632	**0.001**	2.256	1.428, 3.565	**<0.001**

PSM, propensity score matching; ECOG, Eastern Cooperative Oncology Group; AFP, A-fetoprotein; TACE, transarterial chemoembolization; PVTT, portal vein tumor thrombus; HVTT, hepatic vein tumor thrombus.

## Data Availability

The data used in the study are available from the corresponding author on reasonable request.
